# Study of Ultrasound-Assisted Low-Pressure Closed Acid Digestion Method for Trace Element Determination in Rock Samples by Inductively Coupled Plasma Mass Spectrometry

**DOI:** 10.3390/molecules30020342

**Published:** 2025-01-16

**Authors:** Xijuan Tan, Yunxiu Ren, Ting Liang, Denghong Wang

**Affiliations:** 1 Laboratory of Mineralization and Dynamics, College of Earth Sciences and Land Resources, Chang’an University, 126 Yanta Road, Xi’an 710054, China; renyunxiu123@163.com (Y.R.); liangt@chd.edu.cn (T.L.); 2 Institute of Mineral Resources, Chinese Academy of Geological Sciences, Beijing 100037, China

**Keywords:** low-pressure closed acid digestion, ultrasound irradiation, rock samples, trace element extraction, ICP-MS

## Abstract

In this paper, a method of ultrasound-assisted low-pressure closed acid digestion followed by inductively coupled plasma mass spectrometry (ICP-MS) analysis was proposed for trace element quantification in rock samples. By using 1.5 mL of a binary acid mixture of HNO_3_–HF with a ratio of 2:1, rock powder samples of 50 mg were completely decomposed in 12 h at 140 °C after 4 h of ultrasonic treatment with or without pressure relief procedure. The element extraction efficiency of this method was evaluated via the yielded relative errors (REs) of the trace elements in a series of geological standard reference materials (SRMs) with compositions from basic to acidic. It was found that the contents of trace elements (i.e., 36 metal elements from Li to U) in basalt BCR-2, diabase W-2a, andesite AGV-2, granodiorite GSP-2, and granite GSR-1 were comparable with the reported reference values, giving REs with absolute values less than 10%. It was also found that clear solutions without sample powder residues by naked-eye observation can be obtained when using the low-pressure closed decomposition method without ultrasonic pretreatment. The quantification results, however, were found to be negatively biased for most of the studied trace elements, and, in particular, the content bias of Zr in SRM GSP-2 was down to −86.28% due to the low extraction efficiency of refractory minerals of the low-pressure closed digestion method. By applying this proposed digestion strategy, the decomposition property of the ternary combination of HNO_3_–HF–mannitol in terms of trace element quantification accuracy was also investigated. Results showed that the concentrations of trace elements in the studied SRMs were consistent with the reference values, giving REs within ±6.94%, which revealed that there was no deterioration of extraction efficiencies of trace elements and neglected mass interferences from mannitol. This study demonstrated the essential role of ultrasound irradiation in rock sample decomposition to achieve the high extraction efficiency of trace elements under a low-pressure environment, and the developed approach with promising future applications in geoscience exhibited considerable merits, including a high extraction efficiency, feasible digestion process, less time consumption, and lower safety associated risks.

## 1. Introduction

Elements in geoscience are qualitatively classified into major, minor, and trace elements, showing concentration levels higher than 0.4%, within a range of 0.1–0.4%, and less than 0.1% by weight, respectively [1]. It is known that major elements are abundant enough to define the primary structure of a given phase, and they are utilized to determine how phase assemblages evolve during the petrogenesis of rocks [1,2]. On the contrary, trace elements occur passively as dissolved constituents or in the form of minor amounts of accessory phases, which, thus, do not directly influence the properties of a given phase built by major elements [3]. Compared to major elements, however, trace elements can provide geochemical and geological information out of proportion to their abundance due to larger concentration variations and simpler behaviors [4,5,6]. As the efficient information carriers in geological activities, trace elements have been of enormous usage in the investigation of the petrogenesis of igneous rocks [7], detrital provenance [8], arc magma evolution [9], and mineralization processes [10,11,12], etc.

Currently, the analysis of multiple trace elements in whole rocks are generally performed by inductively coupled plasma mass spectrometry (ICP-MS) [13,14] due to the advantages of wide dynamic ranges, low detection limits and high sensitivities [15]. But the strategy of whole-rock ICP-MS analysis undoubtedly involves a prior sample preparation to dissolve the interested components in an aqueous form, making sample decomposition become a critical step and a significant limiting factor in geochemical study. Complete sample digestion is therefore the single most essential prerequisite for accurate trace element quantification [16,17], and a favored sample digestion method was supposed to meet the requirements, including the shortest decomposition time, low reagent consumption, and minimal waste generation [18]. Generally, alkaline fusion and acid digestion are two commonly utilized digestion methods for various geological samples [19,20]. Despite being an efficient and reliable approach to decompose the acid-resistant minerals (e.g., zircon, garnet, barite, and spinel, etc.) in rock samples, alkaline fusion presents relatively high blank and high total dissolved solids (TDSs) in the final solution [21,22]. Such drawbacks make the alkaline fusion method unfavorable in the application of trace element quantification in geological samples. Acidic digestion, decomposing samples via different acid combinations of hydrofluoric acid (HF) with nitric acid (HNO_3_), hydrochloric acid (HCl) and/or perchloric acid (HClO_4_), etc. [23], is known to be capable of decomposing most mineral matrices including Si-O bonds, showing analytical merits of low matrix interferences and improved sensitivities [24,25]. By applying the acid digestion method, sample decomposition can be accomplished in closed high-pressure heated polytetrafluoroethylene (PTFE) bombs [26,27], or in closed screwcap polyfluoroalkoxy (PFA) vessels via low-pressure heating or microwave oven treatment [28,29]. But the latter was not recommended for routine usage even for basic rocks due to less efficiency of the total sample digestion [30,31]. In view of high mineral digestion efficiency, zero loss of volatile elements, and low reagent consumption, acid digestion using closed high-pressure heated bombs has been a favored laboratory sample digestion technique [26,31]. However, this method suffers from the labor-intensive, corrosive issue of bomb-sealed metal jackets and the potential risk of contamination from the corroded metal device [26,32].

It is known that ultrasound irradiation can pass through the medium and cause acoustic cavitations with a microbubble formation and an implosion when imparted to solutions [33,34]. The collapse of bubbles during sonication treatment leads to the generation of the extremely high local temperature of about 5000 K and pressure gradients within 50–1000 atm [35], and the yielded localized “hot spots” can release large amounts of energy [36]. Furthermore, the reactivity of some chemicals can be increased by ultrasound irradiation [37]. These specifically mechanical and chemical properties of ultrasound cause effective ruptures of solid particles, thus causing enhanced solid–liquid leaching [38,39]. The ultrasound-assisted extraction technique combining acidic digestion has been reported for various element analysis in different sample matrices, such as fly ash [40,41], coal [42], rock [43,44,45,46,47,48,49], soil, and sediment [50,51,52]. According to the literature, the extraction efficiency of ultrasound irradiation for trace elements in geological samples, including basaltic rock and carbonatite rock, were investigated. For example, Verni et al. [46] applied a one-step ultrasound-assisted acidic digestion method for about 0.05 g of basic volcanic rock samples using 2 mL of HNO_3_ and 1 mL of HF and successfully obtained the profiling of rare earth elements. However, the occasional manual stirring was required during ultrasound treatment. Furthermore, the absolute values of relative errors (REs) evaluated by two soil reference materials were as high as 36% for the element Sm and 82% for the element Dy. Similarly, despite Diehl et al. [47] optimizing ultrasound-assisted extraction parameters, the highest extraction efficiency of the rare earth elements in carbonatite rocks was found to be about 82%. The authors thus concluded that ultrasound was feasible to improve the leaching efficiency of rare earth elements from carbonatite rocks, but a careful study was necessary if the sample batch was on an industrial scale. Collectively, the ultrasound-assisted digestion technique was limited in the application of trace element quantification in a certain number of geological samples due to relatively low extraction efficiency. To the best of our knowledge, there has been no study on trace element extraction efficiency from geological samples by incorporating the ultrasound treatment into the low-pressure heating acidic digestion method.

In this current work, a low-pressure heating acidic digestion method coupled with the ultrasound pretreatment for rock sample decomposition with the quantification accuracy of 36 trace elements from Li to U by ICP-MS was investigated in detail for the first time. The decomposition efficiency of this ultrasound-assisted low-pressure acidic digestion method was evaluated via REs of the obtained trace elements in a series of geological standard reference materials (SRMs) with chemical compositions from basic to acidic. The determination results of the standard materials were also compared to those by using the proposed method with an extra pre-pressure relief process and the direct low-pressure heating method, which skipped ultrasonic pretreatment. Furthermore, by applying this ultrasound-assisted low-pressure heating digestion strategy, the decomposition property of a ternary mixture of HNO_3_–HF–mannitol was studied based on the accuracy evaluation of trace element quantification.

## 2. Results and Discussion

### 2.1. Extraction Property of Ultrasound-Assisted Low-Pressure Closed Digestion Method

Since the binary acid mixture of HNO_3_ and HF is a desired combination to achieve the complete decomposition of rock samples under high-pressure closed conditions [26], HNO_3_ and HF were utilized as the default digestion reagents to evaluate the ultrasound-assisted low-pressure closed digestion method in this work. The rock samples immersed in the acid mixture were tightly sealed in PFA vials after the pressure relief procedure. The sample PFA vials were then put into a designed glass container for ultrasonic treatment. By taking the complex composition of geological samples [53] and the energy degradation when ultrasonic irradiation passes through the walls of the glass container and the PFA vials [25] into consideration, the output amplitude and temperature of the ultrasonic bath were set as 100%, and 80 °C, respectively. To reduce the influence of ultrasonic intensity differences on the sample decomposition [54], the glass container with deposited sample vials was positioned in the middle of the ultrasonic bath in this current work.

**Figure 1:**
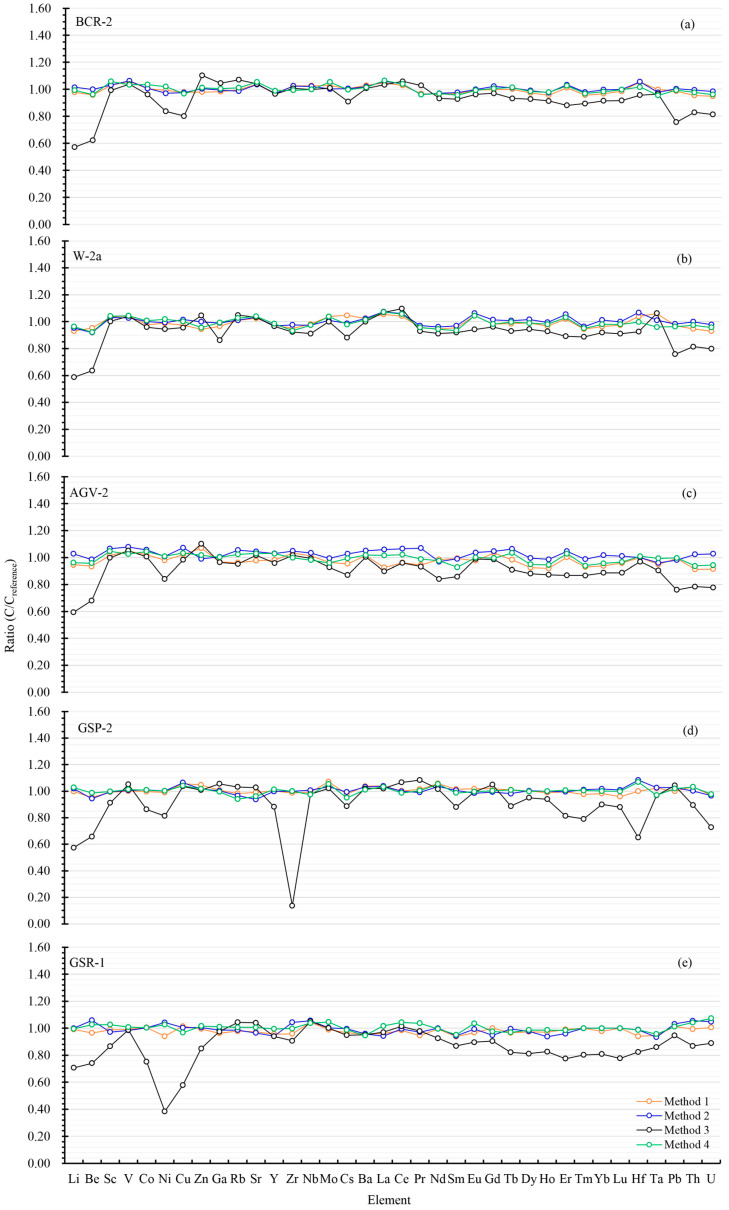
Ratios of quantified contents of trace elements to reference values in silicate SRMs by different digestion methods. Here, five silicate standard materials including BCR-2 (**a**), W-2a (**b**), AGV-2 (**c**), GSP-2 (**d**), and GSR-1 (**e**) were applied in this study. For each method, four parallel specimens of one silicate standard material with a sample weight of 50 mg (±0.5 mg) were digested. The trace element contents utilized to yield the ratios were the average of ICP-MS quantification results of the four specimens.

By following the digestion process of the ultrasound-assisted low-pressure method (Method 1, see digestion method description below), trace elements from Li to U in a series of frequently utilized silicate SRMs with basic to acidic compositions, including BCR-2 (basalt, Columbia River, Oregon), W-2a (diabase, Bull Run quarry, Virginia), AGV-2 (andesite, Guano Valley, Oregon), GSP-2 (granodiorite, Silver Plume, Colorado) [55], and GSR-1 (granite, Binzhou, Hunan Province) [56,57], were measured by ICP-MS. The quantification results in the form of 2σ are summarized in Table 1 and Table S1. By comparing to the reported element mass fractions, the yielded ratios for the studied five SRMs were well within 0.91–1.07 (see Figure 1). From Table 1 and Table S1, the REs (i.e., relative errors) were found to be in the range of −5.04–5.83% for BCR-2, −7.01–6.02% for W-2a, −8.68–6.85% for AGV-2, −4.61–6.99% for GSP-2, and −6.29–4.40% for GSR-1, respectively. Apparently, the absolute values of the REs were less than 10%, demonstrating that the extraction of trace elements from these SRMs by this proposed digestion method was complete or near-total. By the utilizing acid combination of HNO_3_-HF as the decomposing reagent for rock samples, the digestion and element extraction efficiency of this developed ultrasound-assisted low-pressure closed digestion method was comparable to that of the conventional high-pressure closed digestion method [26], making this proposed method a promising alternative strategy in laboratory rock sample digestion.

### 2.2. Pressure Relief Study of the Proposed Ultrasound-Assisted Low-Pressure Closed Digestion Method

It is known that the pressure relief before rock sample digestion, which is designed to partially release the formed volatile SiF_4_ [24], is routinely incorporated in a high-pressure closed acid digestion method due to the potential safety issue from the pressure of SiF_4_ in a sealed bomb with limited space [26]. It can be deduced that the pressure relief might not be a pivotal procedure under low-pressure heating conditions for rock sample digestion. In this work, the effect of pressure relief on the element extraction efficiency of the proposed ultrasound-assisted low-pressure digestion method was thus assessed in detail.

The rock samples were fortified with 1.0 mL of HNO_3_ and 0.5 mL of HF and then directly decomposed using Method 2, which skipped the pressure relief step described in Method 1. The quantification results of trace elements in the five SRMs are collected in Table 1 and Table S1. It is observed that there were no significant differences in trace element concentrations obtained by using Method 1 and Method 2, showing that the pressure relief did not exhibit obvious influence on the digestion efficiency of this ultrasound-assisted low-pressure closed digestion method for rock samples. It is also clear that the values of REs were from −6.64% to 8.28% (see Table 1 and Table S1), with the yielded ratios of the obtained concentrations by this method to reference values in a range of 0.92–1.08 (see Figure 1). This revealed that the pressure relief step was not a necessity in an ultrasound-assisted low-pressure closed acid digestion method, and the sample treating procedures in the proposed Method 1 can thus be simplified to that in Method 2, which shortened the total digestion time at least 2 h.

### 2.3. A Comparison of the Digestion Efficiency to Traditional Low-Pressure Closed Digestion Method

To further assess the effect of ultrasonication on rock sample digestion in the proposed ultrasound-assisted low-pressure closed acid digestion method, the rock samples were treated using a traditional low-pressure closed acid digestion method without ultrasonic treatment (Method 3). After having been heated directly on a hotplate at 140 °C for 12 h, clear sample solutions without any sample powder residues were obtained by naked eye observation. As can be seen in Table 1, the contents of the trace elements generally showed negative bias, yielding ratios of the obtained concentrations to the reference values down to 0.14 (see Figure 1).

**Figure 2:**
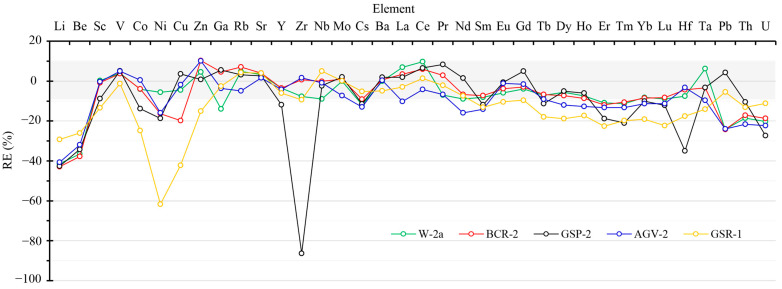
Results of REs of trace element quantification by using the low-pressure closed acid digestion method. Here, five silicate standard materials including BCR-2, W-2, AGV-2, GSP-2, and GSR-1 of 50 mg (±0.5 mg) were decomposed by the low-pressure closed acid digestion method without ultrasound sonication treatment. The values of REs were obtained by the math calculation form of (C − C_reference_)/C_reference_ × 100%.

The obtained REs for trace elements in the studied SRMs by using Method 3 as the digestion method are graphically shown in Figure 2. Despite the yielded REs varying with standard materials and elements, it can be seen from Figure 2 that the values of REs were below zero for most of the studied trace elements. This demonstrated that the traditional low-pressure closed acid digestion method failed to completely extract the trace elements from rock samples, thus resulting in low content recoveries. On the other hand, the negatively biased results should be ascribed to the low extraction efficiency of trace elements under the current low-pressure heating condition. It was also found that the RE value of the element Zr in SRM GSP-2, which is one of the typical refractory geological samples with Zr content high as 550 ± 30 µg/g [26,58], was specifically low and reached −86.28%. Such an extremely negative bias reconfirmed that the conventional low-pressure closed acid digestion method was not recommended to be applied in the decomposition of rock samples containing refractory minerals [25]. Here, it is worth noting that elements with the lowest masses (i.e., Li and Be) and highest masses (i.e., Th and U) exhibited obviously lower values of REs for all the rock reference materials irrespective of composition property. However, the reason causing such a phenomenon remains unclear. Collectively, it is no doubt that ultrasound irradiation can significantly enhance the decomposing and element-extracting abilities of the low-pressure acidic digestion method for rock samples, in particular, the samples containing refractory minerals.

### 2.4. Trace Element Quantification Accuracy of the Proposed Method Based on HNO_3_–HF–Mannitol

According to our previous work, it was found that the HNO_3_–HF–mannitol trinary mixture was desirable for element B quantification in silicate samples via the formation of B-mannitol complex under low-pressure heating conditions with the digestion temperature less than or equal to 140 °C [59]. However, there has been no study of the digestion efficiency of this organic reagent mannitol on trace element quantification. In this work, the proposed ultrasound-assisted low-pressure method with the mixture of HNO_3_–HF–mannitol as the decomposing reagent for trace element quantification in rock samples was investigated. With these five SRMs decomposed using Method 4, the trace elements were determined by ICP-MS, and the content results are listed in Table 1 and Table S1. The concentration levels of the trace elements in the studied SRMs were found to be comparable with those obtained by using Method 1 and Method 2, giving the values of REs within ±10% (see Figure 1). This demonstrated that there was no deterioration of extraction efficiencies of trace elements and neglected mass interferences from mannitol. Hence, it can be inferred that this HNO_3_–HF–mannitol-based ultrasound-assisted low-pressure digestion method can be employed to accurately quantify both trace elements from Li to U and the element B in silicate rock samples simultaneously. This highly promised the future applications of element B together with other trace elements in geological studies.

## 3. Materials and Methods

### 3.1. Apparatus for Ultrasonication

In this current work, all the experiments involving ultrasound treatment were performed by using a KQ-500DE ultrasound bath (Kunshan Ultrasonic Instrument Co., Ltd., Kunshan, China). To preclude the occurrence of the upside down of the digestion vessels during ultrasonic irradiation, a glass container was specifically designed for the allocation of sample digestion vials. This ultrasound bath can provide the highest frequency of 40 kHz and a nominal power of 500 W. The output amplitude and temperature of this utilized ultrasound bath can be operated in a range of 40–100% and 10–80 °C, respectively. Such an ultrasonic apparatus is capable of continuously working 8 h with the heating temperature controlled within ±5 °C.

### 3.2. ICP-MS Instrumentation

Trace element measurements were carried out on a single quadrupole ICP-MS (Agilent 7900 ICP-MS, Santa Clara, CA, USA). This ICP-MS instrument is equipped with a shielding combination of a Pt plate/silicon cap to enhance the element signal sensitivities, an assemble of Ni sampling/skimmer cones (1.0/0.45 mm), a hyperbolic quadrupole mass analyzer, and an orthogonal detector. The detailed description of this instrument was given in our previous work [59,60].

A standard forward power of 1550 W was afforded to the working ICP-MS instrument, and a default autotune process was conducted to examine the instrumental status after warmup. In brief, the torch axis, EM, plasma, off-axis ion lens, and mass resolution/axis were automatically tuned using a tuning solution, which contained 1.0 ng/mL of Li, Y, Ce and U, with the plasma/auxiliary/nebulizer gas flows set at 15/1.0/1.0 L/min. A successful autotune was achieved with the oxide formation (CeO^+^/Ce^+^) and doubly charged species (Ce^2+^/Ce^+^) lower than 2.0%, and the bias of mass axis for Li, Y and U within ±0.05. The pulse and analog modes of the detector were then refreshed with the P/A factor calibrated using 50 ng/mL of multi-element solution, which consisted of all the targeted elements in this study.

To obtain the highest possible sensitivities for low to high-mass isotopes for the instrument, a method tuning with sampling depth, nebulizer gas flow, and the voltages of ion extract, omega bias, omega lens, and deflect were carried out using 1.0 ng/mL of tuning solution before any quantification. Thereafter, the whole ICP-MS system was flushed using a solution of digested silicate sample for at least 30 min. When the measurement started, a standard solution of 50 ng/g was repeatedly quantified every five unknown samples to monitor instrument drifting. The potential memory effect was circumvented by continuously washing the system in a 2% HNO_3_ (*v*/*v*) solution between two adjacent measurements, with the signal intensity recovery of the internal standard element Rh of 25 ng/mL checked. The element determination was carried out under no gas mode of ICP-MS, and the data were read using the peak jumping mode with the isotope dwell time set at 0.3 s. The typical operating parameters for this ICP-MS in this current work are summarized in Table 2.

### 3.3. Chemicals and Reagent Preparation

To reduce the procedure blanks in trace element quantification, ultrapure chemical reagents involving acids and water were utilized throughout the work. Here, the commercially available acids in AR grade including HNO_3_ (68%, *v*/*v*) and HF (40%, *v*/*v*) were purified using sub-boiling distillation systems (Savillex DST-1000-PFA, Eden Prairie, MN, USA) to remove metallic or cationic residues before usage. The ultrapure water with a resistivity of 18.2 MΩ·cm was achieved by using a Milli-QR EQ 7000 water purification system (Millipore, Bedford, MA, USA).

Five external calibrators with concentrations of 5, 25, 50, 100, and 200 ng/mL in 2% HNO_3_ solution (*v*/*v*) were prepared progressively by a gravimetric dilution method from Multi-element Calibration Standard solutions of 100 μg/mL (Agilent Technologies, Tokyo, Japan). The 2% mannitol (*wt.*) solution was prepared by dissolving 1.6 g of mannitol (the China National Pharmaceutical Group Co., Ltd. (Shanghai, China), AR grade) in ultrapure water with a final solution weight of 80 g. The Rh solution with a concentration of 500 ng/g as the online internal standard was prepared by diluting 50 μL of 1.0 mg of the mono-element Rh standard solution (the National Institute of Standards and Technology, Beijing, China) to 100 g using 2% HNO_3_ solution (*v*/*v*).

### 3.4. Silicate Standard Materials

The digestion property and trace element analytical accuracy in this current work were carried out on five silicate standard reference materials from basic to acidic compositions, which have been frequently used in geological associated studies. These standard reference materials included the basic basalt BCR-2 and diabase W-2a, the intermediate andesite AGV-2 and granodiorite GSP-2, and the acidic granite GSR-1. Here, the BCR-2, W-2a, AGV-2 and GSP-2 are geochemical standard reference materials from the Geological Survey of the United States (U.S.), with the preferred values as a reference taken from the GeoReM database [55]. The GSR-1 is a Chinese national igneous rock standard material with detailed information given by Xie et al. [56,57].

### 3.5. Digestion Method Description

In this work, the sample digestion was finished in a Class 1000 clean room. To preclude any contaminants from labware, the utilized PFA vials were heated at 120 °C in aqua regia (the mixture of HNO_3_ and HCl with a ratio of 1:3, *v*/*v*) for 24 h and subsequently in ultrapure water for another 24 h. Thereafter, the PFA vials were carefully rinsed three times with ultrapure water and let dry before usage. Rock samples of 50 mg (±0.5 mg) were weighed in 10 mL of PFA vials and then digested following Methods 1–4, with procedures briefly summarized in Table 3.

Method 1: (1) With 0.5 mL of HNO_3_ and 0.3 mL of HF added, the samples were heated to incipient dryness at 140 °C. (2) Then, 0.5 mL of HNO_3_ and 0.2 mL of HF were added, and the tightly sealed vials were placed in an ultrasonic bath for pretreatment 4 h. (3) The vials were then put back on the hotplate and heated for 12 h at 140 °C. (4) With samples evaporated to incipient dryness, 1 mL of HNO_3_ was added and dried again. Then, 2.0 mL of 40% HNO_3_ was added, and the samples were fluxed over 4 h. (5) After aging overnight, the solutions were transferred to polyethylene terephthalate (PET) bottles and then gravimetrically diluted to 50 ± 0.5 g using a 2% HNO_3_ solution (*v*/*v*).

Method 2: (1) With 1.0 mL of HF and 0.5 mL of HNO_3_ added, the vials were tightly sealed and placed in an ultrasonic bath for pretreatment 4 h. (2) The vials were then transferred on the hotplate and heated for 12 h at 140 °C. (3) With samples evaporated to incipient dryness, 1 mL of HNO_3_ was added and dried again. Then, 2.0 mL of 40% HNO_3_ was added, and samples were fluxed over 4 h. (4) After aging overnight, the solutions were transferred to PET bottles and then gravimetrically diluted to 50 ± 0.5 g using a 2% HNO_3_ solution (*v*/*v*).

Method 3: (1) With 1.0 mL of HF and 0.5 mL of HNO_3_ added, the vials were tightly sealed and heated for 12 h at 140 °C on a hotplate. (2) The samples were then evaporated to incipient dryness and added with 1 mL of HNO_3_. When the second incipient dryness was obtained, 2.0 mL of 40% HNO_3_ was added. (4) After being fluxed over 4 h and aging overnight, the solutions were transferred to PET bottles and then gravimetrically diluted to 50 ± 0.5 g using 2% HNO_3_ solution (*v*/*v*).

Method 4: (1) With 0.6 mL of HF, 30 μL of HNO_3_, and 50 μL of 2% mannitol added, the vials were tightly sealed and placed in an ultrasonic bath for pretreatment 4 h. (2) The vials were then transferred to be heated for 12 h at 140 °C on a hotplate. (3) With samples evaporated to incipient dryness, the samples were added with 1 mL of HNO_3_ and let dry again. Then, 2.0 mL of 40% HNO_3_ was added, and the samples were fluxed over 4 h. (4) After aging overnight, the solutions were transferred to PET bottles and then gravimetrically diluted to 50 ± 0.5 g using a 2% HNO_3_ solution (*v*/*v*).

The final sample solutions from the four methods were taken for trace element quantification by ICP-MS directly. Here, the ultrasound treatment was performed with the input power and temperature set as 100% and 80 °C, respectively.

## 4. Conclusions

In this work, an ultrasound-assisted low-pressure acid digestion method was proposed for accurate trace element quantification in rock samples with basic to acidic compositions. By using 1.5 mL of the HNO_3_–HF mixture with a ratio of 2:1, 50 mg of a series of silicate SRMs (including BCR-2, W-2a, AGV-2, GSP-2, and GSR-1) can be completely decomposed in 12 h at 140 °C following 4 h of ultrasound sonication with or without pressure relief. The ICP-MS quantification results of trace elements were observed to be comparable with the referred mass fractions, yielding the absolute values of REs lower than 10%. It was also found that there were no significant differences in the trace element contents for this developed digestion strategy with or without pressure relief, giving ratios of the obtained concentrations to reference values in a range of 0.91–1.08. Thus, the ultrasound-assisted low-pressure acid digestion method skipping the pressure relief procedure is preferred in rock sample digestion for trace element quantification.

When using the low-pressure decomposition method without the assist of ultrasonic treatment, clear solutions of the SRMs without sample powder residues can be obtained from naked-eye observation. However, the negative bias for the quantification results revealed that this digestion method was unable to completely extract the trace elements from rock samples. The further data analysis showed that the RE value of element Zr in GSP-2 reached −86.28%, confirming that this low-pressure closed acid digestion method was not capable of decomposing rock samples, which contain refractory minerals. Thus, ultrasound pretreatment is highly recommended to be incorporated into the conventional low-pressure acid digestion method to enhance its decomposing ability.

By applying this proposed ultrasound-assisted low-pressure digestion strategy, the decomposition property of a ternary mixture of HNO_3_–HF–mannitol for rock samples was investigated. It was found that the analytical results of trace elements in the studied SRMs agreed with the reference values, giving REs within ±6.94%. Thus, the addition of mannitol did not deteriorate the extraction efficiency of this proposed method and had no obvious mass interferences on ICP-MS analysis, promising the possible application to the accurate quantification of trace elements from Li to U and element B simultaneously.

This in-depth method study revealed that the low-pressure acid digestion method can be applied to rock sample decomposition with trace elements accurately quantified when coupled to ultrasound irradiation. Such a developed ultrasound-assisted low-pressure acid digestion approach exhibits considerable advantages of complete digestion, feasible digestion process, less time consumption and lower safety risks, making it a promising digestion protocol for rock sample preparation in geological laboratory.

## Figures and Tables

**Table 1:** Results of trace element determination with different digestion methods ^1^.

Sample	BCR-2								
Element	Method 1		Method 2		Method 3		Method 4		Reference μg/g
	Content μg/g	RE ^2^ %	Content μg/g	RE %	Content μg/g	RE %	Content μg/g	RE %	
Li	8.92 ± 0.11	−2.25	9.27 ± 0.05	1.51	5.21 ± 0.15	−42.90	9.06 ± 0.10	−0.75	9.13 ± 0.22
Be	2.08 ± 0.01	−4.20	2.17 ± 0.13	−0.14	1.35 ± 0.08	−37.70	2.08 ± 0.10	−3.95	2.17 ± 0.1
Sc	34.53 ± 0.14	3.00	34.60 ± 0.26	3.19	33.27 ± 0.23	−0.78	35.47 ± 0.39	5.79	33.53 ± 0.4
V	441.9 ± 1.7	5.83	443.8 ± 2.5	6.27	433.4 ± 1.4	3.79	431.9 ± 4.9	3.41	417.6 ± 4.5
Co	37.55 ± 0.31	0.58	37.58 ± 0.15	0.66	35.86 ± 0.17	−3.94	38.61 ± 0.39	3.43	37.33 ± 0.38
Ni	12.48 ± 0.22	−0.74	12.20 ± 0.29	−2.93	10.51 ± 0.58	−16.36	12.82 ± 0.15	2.03	12.57 ± 0.37
Cu	19.23 ± 0.30	−2.19	19.19 ± 0.27	−2.38	15.78 ± 0.20	−19.76	19.01 ± 0.37	−3.29	19.66 ± 0.72
Zn	126.9 ± 0.78	−1.97	130.1 ± 0.75	0.43	142.9 ± 0.17	10.34	131.0 ± 1.63	1.19	129.5 ± 1.8
Ga	21.68 ± 0.52	−1.78	21.96 ± 0.18	−0.51	23.08 ± 0.18	4.58	22.17 ± 0.21	0.45	22.07 ± 0.19
Rb	45.82 ± 0.21	−0.43	45.39 ± 0.35	−1.37	49.28 ± 0.11	7.09	46.57 ± 0.45	1.19	46.02 ± 0.56
Sr	350.6 ± 3.2	3.91	349.5 ± 3.6	3.60	350.9 ± 1.6	3.99	355.7 ± 5.3	5.5	337.4 ± 6.7
Y	34.97 ± 0.26	−3.04	35.01 ± 0.39	−2.97	34.81 ± 0.20	−3.48	35.69 ± 0.38	−1.05	36.07 ± 0.37
Zr	191.5 ± 1.62	2.67	191.1 ± 1.21	2.46	187.7 ± 1.12	0.63	185.1 ± 1.49	−0.75	186.5 ± 1.5
Nb	12.76 ± 0.05	2.60	12.72 ± 0.12	2.21	12.43 ± 0.06	−0.08	12.43 ± 0.13	−0.11	12.44 ± 0.2
Mo	257.6 ± 7.1	2.78	251.1 ± 6.9	0.21	253.1 ± 13.5	1.01	263.9 ± 10.8	5.33	250.6 ± 6.7
Cs	1.17 ± 0.01	0.47	1.17 ± 0.01	0.61	1.05 ± 0.01	−9.09	1.16 ± 0.01	−0.25	1.16 ± 0.02
Ba	704.4 ± 6.9	2.99	697.8 ± 5.9	2.03	688.8 ± 2.8	0.71	694.0 ± 4.8	1.47	683.9 ± 4.7
La	26.24 ± 0.15	4.64	26.58 ± 0.23	5.97	25.94 ± 0.21	3.42	26.71 ± 0.24	6.49	25.08 ± 0.16
Ce	54.65 ± 0.36	2.88	55.17 ± 0.06	3.86	56.27 ± 0.19	5.93	55.17 ± 0.17	3.85	53.12 ± 0.33
Pr	6.60 ± 0.15	−3.31	6.57 ± 0.03	−3.75	7.02 ± 0.02	2.89	6.56 ± 0.04	−3.94	6.827 ± 0.04
Nd	27.29 ± 0.15	−3.44	27.43 ± 0.19	−2.93	26.37 ± 0.11	−6.69	27.38 ± 0.31	−3.12	28.26 ± 0.37
Sm	6.34 ± 0.06	−3.19	6.39 ± 0.02	−2.38	6.08 ± 0.04	−7.19	6.27 ± 0.06	−4.28	6.547 ± 0.02
Eu	1.98 ± 0.02	−0.29	1.99 ± 0.01	−0.16	1.91 ± 0.01	−3.74	1.98 ± 0.01	−0.69	1.989 ± 0.05
Gd	6.79 ± 0.09	−0.28	6.96 ± 0.05	2.18	6.61 ± 0.04	−2.91	6.86 ± 0.05	0.68	6.811 ± 0.08
Tb	1.08 ± 0.01	0.11	1.09 ± 0.02	1.14	1.00 ± 0.01	−6.69	1.09 ± 0.01	1.32	1.077 ± 0.03
Dy	6.26 ± 0.05	−2.58	6.37 ± 0.03	−0.90	5.96 ± 0.05	−7.29	6.33 ± 0.07	−1.52	6.424 ± 0.06
Ho	1.26 ± 0.01	−4.32	1.28 ± 0.01	−2.54	1.20 ± 0.01	−8.62	1.28 ± 0.01	−2.24	1.313 ± 0.01
Er	3.71 ± 0.03	1.21	3.79 ± 0.01	3.37	3.23 ± 0.03	−11.92	3.77 ± 0.04	2.70	3.67 ± 0.04
Tm	0.51 ± 0.01	−4.27	0.52 ± 0.01	−2.20	0.48 ± 0.01	−10.52	0.52 ± 0.01	−3.12	0.5341 ± 0.01
Yb	3.28 ± 0.04	−3.31	3.38 ± 0.03	−0.29	3.10 ± 0.02	−8.52	3.33 ± 0.02	−1.76	3.392 ± 0.04
Lu	0.50 ± 0.01	−1.44	0.50 ± 0.04	−0.19	0.46 ± 0.01	−8.28	0.50 ± 0.02	−0.26	0.5049 ± 0.01
Hf	5.24 ± 0.06	5.45	5.25 ± 0.03	5.59	4.76 ± 0.05	−4.35	5.06 ± 0.05	1.71	4.972 ± 0.03
Ta	0.78 ± 0.01	−0.21	0.77 ± 0.01	−2.29	0.76 ± 0.01	−3.41	0.75 ± 0.01	−4.49	0.785 ± 0.02
Pb	10.44 ± 0.05	−1.38	10.63 ± 0.33	0.42	8.02 ± 0.30	−24.28	10.53 ± 0.17	−0.58	10.59 ± 0.17
Th	5.57 ± 0.06	−4.48	5.80 ± 0.05	−0.44	4.83 ± 0.03	−17.06	5.69 ± 0.05	−2.32	5.828 ± 0.05
U	1.60 ± 0.02	−5.04	1.66 ± 0.01	−1.52	1.37 ± 0.01	−18.65	1.62 ± 0.01	−3.91	1.683 ± 0.02

^1^ Results are given in μg/g and 2σ between 5 and 8 individual ICP-MS analyses of each sample. ^2^ RE is the relative error calculated by the math form of (C − C_reference_)/C_reference_ × 100.

**Table 2:** Operating parameters for ICP-MS in this work ^1^.

Instrument Parameter	Operating Condition	Instrument Parameter	Operating Condition
Spray chamber	Scott chamber at 2 °C	Extract 1, V	0
RF power, W	1550	Extract 2 *, V	−190
Plasma gas, L/min Ar	15.0	Omega bias *, V	−95
Auxiliary gas, L/min Ar	1.0	Omega lens *, V	9.1
Nebulizer gas, L/min Ar	1.05	Discriminators *, mV	3.8
Sample/skimmer cone, mm	Nickle, 1.0/0.45	Analog HV *, V	2221
Sampling depth *, mm	9.0	Pulse HV *, V	1258
Dwell time, s	0.3	Detector mode	Dual
Readings/replicate	5	Scan mode	Peak jumping

^1^ The parameters marked with * are default values, which were optimized by daily method tuning.

**Table 3:** Different low-pressure acidic digestion methods for rock samples.

Procedure	Method			
	Method 1	Method 2	Method 3	Method 4
Pressure relief	Add 0.5 mL of HNO_3_ and 0.3 mL of HF into 50 mg of sample, then dry at 140 °C	–	–	–
Ultrasound-assisted decomposition	Add 0.5 mL of HNO_3_ and 0.2 mL of HF, then place in an ultrasonic bath for 4 h	Add 1.0 mL of HNO_3_ and 0.5 mL of HF into 50 mg of sample, then place in an ultrasonic bath for 4 h	–	Add 30 μL of HNO_3_, 0.6 mL of HF, and 50 μL of 2% mannitol into 50 mg of sample, then place in an ultrasonic bath for 4 h
Low-pressure hotplate digestion	Flux 12 h at 140 °C	Flux 12 h at 140 °C	Add 1.0 mL of HNO_3_ and 0.5 mL HF into 50 mg of sample, then flux 12 h at 140 °C	Flux 12 h at 140 °C
Excess HF removal	When incipient dry was obtained, add 1 mL of HNO_3_ and heat to dry again			
Sample redissolution	Add 2.0 mL of 40% HNO_3_ (*wt.*) and flux over 4 h			
Solution for ICP-MS	Age overnight, and dilute to 50 g using 2% HNO_3_ (*v*/*v*)			

## Data Availability

Data are contained within this article.

## Supplementary Materials

The following supporting information can be downloaded at https://www.mdpi.com/article/10.3390/molecules30020342/s1, Table S1: Results of trace element determination with different digestion methods.
