# Supplementary material for: Study of Ultrasound-Assisted Low-Pressure Closed Acid Digestion Method for Trace Element Determination in Rock Samples by Inductively Coupled Plasma Mass Spectrometry
Source: Molecules. 2025 Jan 16;30(2):342. doi: 10.3390/molecules30020342 (PMC11767439; doi:10.3390/molecules30020342)
Supplement: Supplementary file 1 [file molecules-30-00342-s001.zip › molecules-3332738-supplementary.pdf]

molecules

MDPI

Article

Study of Ultrasound-Assisted Low-Pressure Closed Acid Digestion Method for Trace Element Determination in Rock Samples by Inductively Coupled Plasma Mass Spectrometry

Xijuan Tan [1,*], Yunxiu Ren [1], Ting Liang [1] and Denghong Wang [2,*]

1 Laboratory of Mineralization and Dynamics, College of Earth Sciences and Land Resources, Chang'an University, 126 Yanta Road, Xi'an 710054, China; renyunxiu123@163.com (Y.R.); liangt@chd.edu.cn (T.L.)
2 Institute of Mineral Resources, Chinese Academy of Geological Sciences, Beijing 100037, China
* Correspondence: tanxijuan@hotmail.com or tanxijuan@chd.edu.cn (X.T.); wangdenghong@vip.sina.com (D.W.)

Supporting information

Table S1. Results of trace element determination with different digestion methods [1].

Sample	W-2a								
Element	Method 1		Method 2		Method 3		Method 4		Reference μg/g
	Content μg/g	RE [2] %	Content μg/g	RE %	Content μg/g	RE %	Content μg/g	RE %	
Li	8.57±0.05	−6.98	8.78±0.07	−4.70	5.41±0.12	−41.31	8.87±0.04	−3.66	9.21±0.19
Be	0.64±0.01	−4.72	0.62±0.04	−8.01	0.43±0.01	−36.44	0.62±0.01	−7.79	0.672±0.048
Sc	37.08±0.12	3.41	36.95±0.51	3.05	35.95±0.36	0.26	37.36±0.38	4.19	35.86±0.38
V	277.4±3.6	4.37	273.1±2.7	2.74	276.7±3.0	4.08	277.3±2.9	4.32	265.8±2.9
Co	43.41±0.38	−2.17	44.42±1.23	0.12	42.58±0.43	−4.04	44.72±0.45	0.79	44.37±0.65
Ni	71.08±0.69	−1.27	71.49±1.90	−0.71	68.02±0.23	−5.53	73.34±0.61	1.85	72±1
Cu	103.1±0.88	−2.60	107.5±2.72	1.48	101.2±1.72	−4.41	106.2±2.10	0.26	105.9±1.5
Zn	73.40±0.91	−5.53	77.64±4.82	−0.08	81.25±1.14	4.57	74.25±0.53	−4.44	77.7±1.6
Ga	17.25±0.13	−3.51	17.71±0.42	−0.92	15.40±0.03	−13.89	17.75±0.15	−0.75	17.88±0.31
Rb	20.42±0.16	0.93	20.45±0.27	1.10	21.22±0.10	4.91	20.78±0.15	2.72	20.23±0.27
Sr	200.4±0.70	2.55	201.8±2.9	3.29	201.8±0.78	3.29	203.1±1.22	3.96	195.4±1.6
Y	21.22±0.11	−2.76	21.13±0.07	−3.18	21.05±0.04	−3.51	21.47±0.26	−1.60	21.82±0.33
Zr	88.58±0.64	−5.06	91.09±2.86	−2.37	86.13±1.55	-7.69	86.99±3.01	−6.76	93.3±1.4
Nb	7.37±0.15	−1.91	7.32±0.03	−2.59	6.84±0.30	−8.89	7.32±0.02	−2.58	7.51±0.15
Mo	0.48±0.01	3.79	0.47±0.03	0.85	0.46±0.04	−0.01	0.48±0.03	3.74	0.465±0.03
Cs	0.96±0.00	4.63	0.90±0.01	−1.28	0.81±0.01	−11.81	0.90±0.01	−2.11	0.915±0.016
Ba	177.1±1.70	2.49	176.8±0.72	2.32	172.9±1.16	0.03	175.2±0.79	1.38	172.8±1.9
La	11.21±0.11	5.48	11.42±0.08	7.39	11.36±0.14	6.90	11.38±0.09	7.10	10.63±0.12
Ce	24.18±0.25	4.19	24.58±0.14	5.91	25.47±0.20	9.75	24.53±0.17	5.68	23.21±0.17
Pr	2.89±0.03	−4.39	2.93±0.02	−2.94	2.81±0.02	−7.02	2.89±0.02	−4.33	3.018±0.033
Nd	12.37±0.08	−5.47	12.59±0.03	−3.83	11.93±0.13	−8.87	12.37±0.15	−5.53	13.09±0.12
Sm	3.15±0.02	−4.67	3.20±0.02	−3.01	3.03±0.01	−8.09	3.08±0.06	−6.66	3.3±0.13
Eu	1.14±0.01	4.43	1.16±0.01	6.28	1.03±0.01	−5.82	1.14±0.01	4.43	1.091±0.011
Gd	3.64±0.02	−1.89	3.77±0.03	1.46	3.57±0.01	−3.91	3.65±0.03	−1.79	3.713±0.039
Tb	0.62±0.01	−1.47	0.63±0.01	0.74	0.58±0.01	−7.11	0.62±0.01	−0.32	0.627±0.008
Dy	3.77±0.03	−0.96	3.87±0.02	1.57	3.59±0.03	−5.56	3.77±0.06	−1.03	3.806±0.029
Ho	0.76±0.01	−3.34	0.79±0.01	−0.50	0.73±0.01	−7.34	0.78±0.01	−1.86	0.7908±0.006
Er	2.25±0.01	2.00	2.33±0.01	5.41	1.97±0.01	−10.85	2.28±0.03	3.12	2.208±0.025
Tm	0.31±0.01	−5.56	0.32±0.01	−3.70	0.29±0.01	−11.27	0.31±0.01	−5.02	0.3315±0.006
Yb	1.98±0.01	−3.55	2.08±0.03	1.11	1.89±0.02	−8.17	2.01±0.03	−1.93	2.054±0.016
Lu	0.30±0.001	−2.63	0.31±0.01	−0.10	0.28±0.01	−8.94	0.30±0.03	−1.95	0.309±0.0034
Hf	2.54±0.03	3.80	2.61±0.06	6.81	2.26±0.04	−7.54	2.44±0.07	−0.34	2.444±0.041
Ta	0.52±0.02	6.02	0.49±0.02	1.09	0.52±0.01	6.25	0.47±0.01	−3.97	0.489±0.014
Pb	7.61±0.16	−2.87	7.69±0.43	−1.81	5.94±0.05	−24.14	7.55±0.37	−3.63	7.83±0.19
Th	2.06±0.06	−5.33	2.18±0.05	−0.04	1.77±0.07	−18.56	2.12±0.03	−2.69	2.179±0.031
U	0.47± 0.01	−7.01	0.49±0.01	−2.20	0.40±0.01	−20.15	0.48±0.01	−4.38	0.5048±0.007

[1] Results are given in μg/g and 2σ for between 5 and 8 individual analyses of each sample. [2] RE is the relative error calculated by the math form of $(C-C_{reference})/C_{reference}\times100$.

Table S1. (Continued).

Sample	AGV-2								
Element	Method 1		Method 2		Method 3		Method 4		Reference μg/g
	Content μg/g	RE %	Content μg/g	RE %	Content μg/g	RE %	Content μg/g	RE %	
Li	10.21±0.16	−5.49	11.09±0.26	2.73	6.40±0.14	−40.70	10.38±0.08	−3.90	10.8±0.21
Be	2.06±0.06	−6.55	2.18±0.05	−1.48	1.51±0.04	−31.86	2.11±0.04	−4.30	2.209±0.066
Sc	13.30±0.14	1.44	13.97±0.41	6.55	13.07±0.09	−0.31	13.70±0.20	4.53	13.11±0.31
V	124.1±1.1	4.73	127.9±2.8	7.89	124.5±0.2	5.06	121.4±5.3	2.48	118.5±1.2
Co	15.79±0.08	2.11	16.32±0.33	5.59	15.54±0.12	0.53	16.14±0.17	4.41	15.46±0.5
Ni	18.48±0.08	−2.07	19.00±0.42	0.70	15.88±0.29	−15.86	19.03±0.16	0.85	18.87±0.41
Cu	52.56±0.33	2.03	55.23±2.51	7.22	50.58±0.42	−1.81	53.16±0.72	3.20	51.51±0.65
Zn	92.64±5.58	6.85	85.82±3.53	−1.02	95.48±0.52	10.13	88.15±6.81	1.67	86.7±1.2
Ga	19.74±0.11	−3.31	20.49±0.40	0.33	19.68±1.96	−3.61	20.44±0.17	0.08	20.42±0.17
Rb	65.19±1.49	−3.84	71.47±2.19	5.43	64.52±1.84	−4.83	69.35±1.35	2.29	67.79±0.66
Sr	643.7±3.7	−2.39	688.4±15.2	4.39	670.3±3.5	1.63	679.4±9.2	3.01	659.5±5.7
Y	18.77±0.22	−1.95	19.70±0.34	2.94	18.34±0.23	−4.18	19.67±0.18	2.77	19.14±0.84
Zr	239.7±1.14	3.32	243.2±4.52	4.85	235.8±0.88	1.64	231.3±2.19	−0.28	232±2.3
Nb	14.27±0.10	1.07	14.59±0.28	3.32	14.01±0.10	−0.79	13.84±0.05	−2.00	14.12±0.22
Mo	1.93±0.06	−3.65	1.99±0.07	−0.72	1.85±0.01	−7.29	1.92±0.03	−4.03	2±0.11
Cs	1.12±0.01	−4.70	1.20±0.03	2.64	1.02±0.01	−13.00	1.16±0.02	−0.75	1.173±0.018
Ba	1150±13	1.39	1190± 25	4.97	1138±4.405	0.39	1154±9	1.76	1134±8
La	35.36±0.53	−7.47	40.46±1.29	5.90	34.31±1.24	−10.21	38.79±1.03	1.51	38.21±0.38
Ce	66.74±1.63	−3.87	73.97± 1.83	6.54	66.56±1.74	−4.14	70.88±1.36	2.08	69.43±0.57
Pr	7.71±0.41	−5.53	8.73±0.33	6.94	7.62±0.21	−6.69	8.06±0.38	−1.24	8.165±0.084
Nd	30.02±0.21	−1.53	29.54±0.87	−3.12	25.65±0.64	−15.87	29.74±0.40	−2.46	30.49±0.47
Sm	5.47±0.03	−0.73	5.45±0.14	−1.00	4.73±0.07	−14.13	5.12±0.07	−7.11	5.509±0.078
Eu	1.52±0.01	−2.43	1.61±0.04	3.67	1.54±0.02	−1.07	1.55±0.02	−0.06	1.553±0.015
Gd	4.83±0.05	3.30	4.89±0.12	4.59	4.60±0.08	−1.58	4.65±0.05	−0.56	4.678±0.064
Tb	0.64±0.01	−1.73	0.69±0.02	6.29	0.59±0.01	−9.07	0.67±0.01	3.33	0.651±0.0073
Dy	3.28±0.03	−7.49	3.53±0.08	−0.49	3.12±0.04	−11.97	3.36±0.02	−5.20	3.549±0.031
Ho	0.63±0.01	−8.03	0.67±0.01	−1.47	0.60±0.01	−12.73	0.64±0.01	−5.56	0.6818±0.0081
Er	1.83±0.01	0.08	1.91±0.04	4.58	1.58±0.01	−13.18	1.88±0.02	2.76	1.825±0.013
Tm	0.24±0.01	−7.05	0.26±0.01	−1.28	0.23±0.01	−13.27	0.25±0.01	−6.09	0.2623±0.0035
Yb	1.55±0.01	−6.21	1.68±0.05	1.67	1.47±0.01	−11.23	1.58±0.01	−4.38	1.653±0.013
Lu	0.24±0.02	−4.22	0.25±0.07	1.06	0.22±0.01	−11.28	0.24±0.05	−3.41	0.2507±0.0033
Hf	5.13±0.04	−0.14	5.14±0.09	0.04	4.97±0.05	−3.16	5.18±0.06	0.91	5.137±0.057
Ta	0.82±0.01	−5.21	0.83±0.02	−3.95	0.78±0.01	−9.56	0.86±0.02	−0.72	0.865±0.019
Pb	13.03±0.15	−0.82	12.88±0.25	−1.95	9.99±0.16	−23.94	13.09±0.17	−0.40	13.14±0.15
Th	5.64±0.07	−8.68	6.32±0.14	2.29	4.84±0.04	−21.56	5.78±0.04	−6.34	6.174±0.063
U	1.73±0.02	−8.47	1.93±0.04	2.64	1.47±0.02	−22.28	1.78±0.02	−5.65	1.885±0.015

Table S1. (Continued).

Sample	GSP-2 [1]								
Element	Method 1		Method 2		Method 3		Method 4		Reference μg/g
	Content μg/g	RE %	Content μg/g	RE %	Content μg/g	RE %	Content μg/g	RE %	
Li	35.92±1.07	−0.22	36.69±0.35	1.92	20.66±0.21	−42.60	36.97±0.96	2.71	36±1
Be	1.43±0.02	−4.61	1.42±0.03	−5.55	0.99±0.03	−34.20	1.48±0.10	−1.36	1.5±0.2
Sc	6.26±0.13	−0.70	6.27±0.10	−0.53	5.74±0.48	−8.83	6.28±0.04	−0.30	6.3±0.7
V	51.98±0.68	−0.04	52.31±0.60	0.60	54.65±0.24	5.09	52.65±0.10	1.25	52±4
Co	7.27±0.04	−0.37	7.37±0.16	0.92	6.30±0.05	−13.74	7.36±0.06	0.83	7.3±0.8
Ni	16.82±0.28	−1.04	17.03±0.22	0.16	13.82±0.10	−18.70	17.02±0.09	0.12	17±2
Cu	45.46±0.87	5.71	45.75±0.40	6.40	44.54±2.59	3.58	44.71±0.90	3.98	43±4
Zn	125.6±7.34	4.65	121.8±1.12	1.53	121.0±0.84	0.83	122.2±2.09	1.80	120±10
Ga	22.15±0.27	0.70	22.07±0.29	0.32	23.21±0.22	5.51	21.90±0.81	−0.44	22±2
Rb	240.6±16.78	−1.78	236.7±8.12	−3.39	252.9±3.29	3.24	230.3±12.42	−6.02	245±7
Sr	237.6±5.5	−1.02	225.2±6.8	−6.18	246.4±4.3	2.68	230.6±5.9	−3.91	240±10
Y	28.02±0.47	0.09	27.96±0.38	−0.13	24.67±0.59	−11.89	28.38±0.51	1.36	28±2
Zr	542.5±5.8	−1.36	550.0±3.4	0.00	75.46±4.9	−86.28	550.0±45.3	0.00	550±30
Nb	26.79±0.24	−0.77	27.19±0.46	0.72	26.38±0.10	−2.31	26.30±0.31	−2.60	27±2
Mo	2.25±0.06	6.99	2.17±0.07	3.30	2.14±0.44	2.07	2.21±0.07	5.42	2.1±0.6
Cs	1.17±0.02	−2.28	1.19±0.01	−0.62	1.06±0.02	−11.37	1.14±0.01	−4.96	1.2±0.1
Ba	1389±12	3.67	1383±16	3.21	1366±11	1.98	1354±8	1.07	1340±44
La	187.1±5.0	3.92	187.0±5.1	3.87	183.6±5.8	1.99	185.9±5.1	3.30	180±12
Ce	410.5±9.9	0.12	409.5±7.7	−0.13	437.1±10.3	6.60	404.7±9.6	−1.30	410±30
Pr	51.77±1.58	1.51	50.57±1.64	−0.84	55.24±1.60	8.32	51.18±1.16	0.35	51±5
Nd	211.5±4.5	5.75	207.1±6.5	3.56	203.1±4.7	1.57	210.5±5.0	5.25	200±12
Sm	27.38±0.60	1.40	27.16±0.86	0.60	23.79±0.54	−11.90	26.65±0.58	−1.30	27±1
Eu	2.34±0.04	1.78	2.26±0.05	−1.57	2.29±0.05	−0.51	2.29±0.04	−0.42	2.3±0.1
Gd	12.23±0.30	1.91	11.91±0.30	−0.74	12.59±0.44	4.94	12.04±0.23	0.30	12±2
Tb	1.69±0.04	0.98	1.64±0.04	−1.88	1.48±0.04	−11.32	1.69±0.04	0.92	1.67±nd
Dy	5.57±0.11	0.06	5.58±0.14	0.27	5.29±0.14	−5.01	5.56±0.12	−0.19	5.57±nd
Ho	0.89±0.02	−1.07	0.90±0.02	−0.17	0.85±0.02	−5.99	0.90±0.02	−0.11	0.9±nd
Er	2.74±0.04	−0.51	2.74±0.05	−0.42	2.23±0.07	−18.73	2.77±0.07	0.67	2.75±nd
Tm	0.28±0.01	−2.42	0.29±0.01	1.17	0.23±0.01	−20.97	0.29±0.01	0.52	0.29±0.02
Yb	1.37±0.06	−1.79	1.41±0.02	1.62	1.25±0.05	−9.99	1.39±0.03	0.03	1.39±nd
Lu	0.17±0.01	−4.09	0.18±0.02	0.92	0.16±0.01	−12.10	0.18±0.01	−0.11	0.18±0.03
Hf	2.83±0.16	0.00	3.06±0.09	8.28	1.84±0.12	−35.01	3.03±0.25	6.94	2.83±1
Ta	0.84±0.02	2.19	0.84±0.02	2.65	0.79±0.02	−3.26	0.79±0.01	−3.08	0.82±nd
Pb	42.01±0.48	0.00	43.17±0.80	2.78	43.80±1.24	4.29	42.79±0.63	1.87	42±3
Th	111.5±2.85	3.26	108.3±1.37	0.23	96.69±2.46	−10.47	111.4±2.13	3.13	108±8
U	2.33±0.08	−3.03	2.32±0.06	−3.39	1.75±0.05	−27.26	2.34±0.05	−2.29	2.4±0.19

[1] The "nd" is defined as no data for confidence interval.

Table S1. (Continued).

Sample	GSR-1								
Element	Method 1		Method 2		Method 3		Method 4		Reference μg/g
	Content μg/g	RE %	Content μg/g	RE %	Content μg/g	RE %	Content μg/g	RE %	
Li	129.9±1.93	−0.86	131.2±1.52	0.15	92.63±0.76	−29.29	130.4±0.92	−0.45	131±5
Be	11.97±0.32	−3.45	13.12±0.26	5.77	9.18±0.37	−25.98	12.73±0.18	2.68	12.4±1.4
Sc	6.03±0.25	−1.21	5.93±0.10	−2.82	5.29±0.15	−13.32	6.26±0.10	2.70	6.1±0.4
V	23.79±0.18	−0.88	23.62±0.06	−1.56	23.69±0.24	−1.29	24.19±0.33	0.80	24±2
Co	3.42±0.10	0.52	3.41±0.14	0.31	2.56±0.08	−24.72	3.41±0.16	0.28	3.4±0.7
Ni	2.16±0.06	−5.92	2.40±0.08	4.21	0.88±0.01	−61.74	2.36±0.06	2.61	2.3±0.8
Cu	3.25±0.05	1.48	3.21±0.43	0.42	1.85±0.15	−42.13	3.09±0.29	−3.33	3.2±0.9
Zn	27.86±0.97	−0.50	28.15±0.58	0.53	23.78±0.41	−15.06	28.45±0.46	1.62	28±3
Ga	18.29±0.17	−3.75	18.75±0.10	−1.32	18.52±0.12	−2.52	19.21±0.03	1.08	19±2
Rb	456.0±43.7	−2.15	459.6±18.5	−1.38	486.1±3.4	4.32	468.8±11.4	0.61	466±17
Sr	103.1±6.4	−2.72	102.3±2.2	−3.45	110.2±1.1	3.99	106.6±3.9	0.61	106±6
Y	59.29±0.68	−4.38	58.30±2.55	−5.96	58.28±1.22	−6.01	61.69±0.67	−0.50	62±5
Zr	95.17±3.9	−4.17	103.7±7.1	4.38	90.06±1.8	−9.32	99.08±3.2	−0.23	99.31±6
Nb	41.76±0.32	4.40	42.23±1.11	5.59	42.01±0.96	5.02	41.54±0.37	3.84	40±3
Mo	3.46±0.10	−1.05	3.51±0.16	0.28	3.50±0.20	0.07	3.66±0.04	4.49	3.5±0.2
Cs	37.14±0.31	−3.28	38.23±0.48	−0.43	36.40±0.43	−5.22	37.91±0.13	−1.27	38.4±1.2
Ba	327.8±9.8	−4.43	329.0±4.7	−4.09	326.2±1.8	−4.90	324.3±1.6	−5.45	343±29
La	51.87±0.28	−3.94	50.88±0.92	−5.78	52.39±2.51	−2.98	54.89±1.76	1.65	54±4
Ce	106.3±8.01	−1.59	107.4±1.31	−0.53	109.5±4.82	1.39	112.7±3.78	4.35	108±7
Pr	12.01±1.20	−5.44	12.34±0.32	−2.87	12.43±0.49	−2.09	13.18± 0.47	3.76	12.7±0.8
Nd	46.75±1.72	−0.53	47.00±1.22	0.00	43.53±1.46	−7.39	46.75±1.09	−0.53	47±4
Sm	9.09±0.87	−6.29	9.15±0.27	−5.71	8.43±0.13	−13.12	9.23±0.23	−4.83	9.7±0.8
Eu	0.82±0.07	−3.32	0.84±0.02	−0.66	0.76±0.01	−10.38	0.88±0.01	3.59	0.85±0.07
Gd	9.32±0.16	0.22	8.82±0.32	−5.19	8.41±0.14	−9.55	9.07±0.27	−2.44	9.3±0.7
Tb	1.59±0.07	−3.38	1.64±0.07	−0.51	1.35±0.03	−17.91	1.60±0.09	−3.12	1.65±0.09
Dy	9.93±0.34	−2.63	9.97±0.12	−2.27	8.28±0.16	−18.81	10.06±0.11	−1.35	10.2±0.4
Ho	1.99±0.09	−2.96	1.92±0.02	−6.26	1.69±0.03	−17.32	2.02±0.01	−1.35	2.05±0.17
Er	6.43±0.20	−1.01	6.24±0.11	−4.00	5.03±0.11	−22.58	6.39±0.04	−1.64	6.5±0.3
Tm	1.04±0.08	−1.89	1.07±0.04	0.94	0.85±0.02	−19.74	1.10±0.01	3.77	1.06±0.09
Yb	7.24±0.07	−2.19	7.43±0.22	0.41	5.99±0.15	−19.11	7.45±0.07	0.68	7.4±0.5
Lu	1.16±0.08	0.87	1.12±0.03	−2.61	0.89±0.03	−22.30	1.11± 0.02	−3.48	1.15±0.09
Hf	3.53±0.12	−5.95	3.71±0.13	−1.12	3.09±0.05	−17.54	3.70±0.10	−1.29	3.75±0.8
Ta	6.81±0.27	−5.46	6.72±0.10	−6.64	6.19±0.20	−14.04	6.89±0.08	−4.33	7.2±0.7
Pb	31.38±0.64	1.23	31.98±1.34	3.16	29.32±0.30	−5.41	31.37±0.86	1.18	31±3
Th	53.64±2.31	−0.66	56.97±2.20	5.50	46.89±1.27	−13.16	56.29±0.97	4.25	54±3
U	18.89±1.23	0.47	19.71±0.42	4.82	16.70±0.63	−11.16	20.17±0.11	7.31	18.8±1.4
